# Predictors of Mortality in Head-Preserving Treatment for Dislocated Proximal Humerus Fractures: A Retrospective Analysis of 522 Cases with a Minimum Follow-Up of 5 Years

**DOI:** 10.3390/jcm12123977

**Published:** 2023-06-11

**Authors:** Lisa Klute, Leopold Henssler, Christian Pfeifer, Arne Berner, Teresa Schneider, Miriam Kobeck, Volker Alt, Maximilian Kerschbaum

**Affiliations:** 1 Clinic of Trauma Surgery, University Medical Center Regensburg, Franz-Josef-Strauss-Allee 11, 93053 Regensburg, Germany; 2 Clinic of Trauma and Hand Surgery, Altötting, Vinzenz-von-Paul-Straße 10, 84503 Altötting, Germany; 3 Clinic of Trauma Surgery, Bad Neustadt, Von-Guttenberg-Straße 11, 97616 Bad Neustadt an der Saale, Germany

**Keywords:** mortality, humerus fractures, proximal humerus, shoulder

## Abstract

Purpose: Proximal humerus fractures (PHFs) are among the most common fractures in elderly patients, but there is still inadequate knowledge about mortality risk factors after such injuries. In order to provide the best possible therapy, individual risk factors have to be considered and evaluated thoroughly. There is still controversy regarding treatment decisions for proximal humerus fractures, particularly for the elderly. Methods: In this study, patient data from 522 patients with proximal humerus fractures were obtained from 2004 to 2014 at a Level 1 trauma centre. After a minimum follow-up of 5 years, the mortality rate was assessed, and independent risk factors were evaluated. Results: A total of 383 patients (out of 522) were included in this study. For our patient collective, the mean follow-up was at 10.5 ± 3.2 years. The overall mortality rate was 43.8% in our respondent group and was not significantly impacted by concomitant injuries. The binary logistic regression model showed an increased risk for mortality by 10% per life year, a 3.9 times higher mortality risk for men and a 3.4 times higher risk for conservative treatment. The most powerful predictor was a Charlson Comorbidity Index of more than 2, with a 20 times higher mortality risk. Conclusions: Outstanding independent predictors of death in our patient collective were serious comorbidities, male patients, and conservative treatment. This patient-related information should influence the process of decision making for the individual treatment of patients with PHFs.

## 1. Introduction

Proximal humerus fractures (PHFs) account for approximately 8% of all fractures, and their incidence has grown significantly over the past 10 years, especially in female patients over 70 years of age [1]. The acute event of a humeral head fracture often leads to a drastic change in the patient’s life situation due to functional independence. There is currently a strong trend toward performing reversed arthroplasty as the treatment of choice in older patients with complicated fracture entities [2,3,4]. Nevertheless, head-preserving procedures such as osteosynthesis or conservative treatment are still one of the most important therapeutic modalities in all age groups, especially in reconstructable fracture situations [4,5,6]. In the case of head preserving, aftercare is often more complex and demanding than joint-replacing treatment options [7,8].

In the realm of clinical research, there exists a wealth of data regarding the mortality risk and predictors of death in proximal femur fractures that have undergone osteosynthesis [9,10]. However, it is desirable to have comparable levels of data available for PHFs, particularly in relation to head-preserving treatment. While a few studies have described the overall mortality risk associated with PHFs [8], encompassing both head-preserving and head-replacing approaches, there remains a notable gap in the literature regarding a specific mortality risk analysis focused solely on head-preserving treatment, particularly within the context of long-term follow-up.

The high prevalence of PHFs highlights their substantial impact on patient lives and the varying treatment modalities available, with a shift toward reversed arthroplasty [2,3,4], while head-preserving approaches remain vital, especially in reconstructable fractures. The controversial debate on the optimal treatment of PHFs remains an important issue yet to be resolved. The need for comprehensive data on mortality risk and predictors, akin to those available for proximal femur fractures [9,10], emphasises the relevance of investigating this aspect in PHFs. By focusing on the mortality rate within the first five years and evaluating the predictors of death after head-preserving surgery, our study aims to contribute valuable insights to optimise patient outcomes and guide clinical decision making.

A prospective randomised controlled trial conducted in the UK stands as a seminal study in Europe concerning proximal humerus fractures (PHFs). This study aimed to compare the outcomes of conservative and surgical treatment approaches for PHFs, and it revealed noteworthy findings. Particularly, the trial observed higher rates of complications and mortality among patients who underwent surgical treatment [8]. However, in terms of functional outcomes, no significant difference was observed between the two treatment groups.

Considering these findings, we formulated a hypothesis that advocates for the potential of conservative therapy to enhance survival rates in PHF patients. Consequently, the primary objective of this study was to thoroughly analyse the mortality rate of PHFs within a long-term follow-up. Our investigation specifically aimed to evaluate the potential predictors of death following both conservative therapy and reconstructive surgery. By conducting this analysis, we sought to bridge the existing knowledge gap and provide valuable insights into the mortality risk and associated factors pertaining to head-preserving treatment for PHFs.

## 2. Materials and Methods

**Figure 1:**
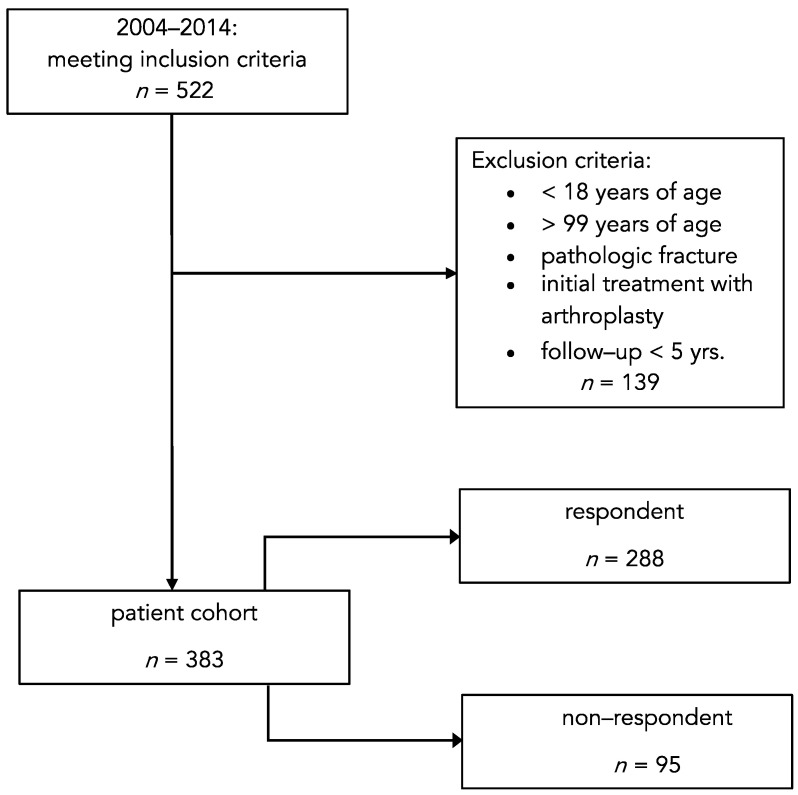
Flowchart of case inclusion and exclusion. The study population only consisted of patients with proximal humerus fractures and a follow-up of at least 5 years.

We conducted a study involving a retrospective identification of patients with proximal humerus fractures who were diagnosed in our trauma department over a period spanning from 2004 to 2014. To ensure the reliability and validity of our findings, we applied specific exclusion criteria. Patients who were below 18 years of age or above 99 years, those with pathologic fractures, individuals with a follow-up duration of less than 5 years, and those who underwent arthroplasty as the primary treatment for their fractures were excluded from the analysis (Figure 1). By implementing these stringent criteria, we aimed to establish a homogeneous patient cohort for our study. Following the application of these parameters, we conducted a prospective patient survey, contacting the remaining eligible individuals from our identified patient cohort. Ultimately, our study included a total of 383 patients who met the inclusion criteria. Among these, 288 patients responded and provided the necessary information for our analysis, while 95 patients did not respond. The response rate of our survey was 75.2% (288 out of 383 patients).

To ensure the ethical soundness of our research, the study protocol and procedures were reviewed and approved by the Ethics Committee of our local university. This approval signifies that our study adhered to the relevant ethical guidelines and regulations governing human subject research.

### 2.1. Parameter Investigation

For our study, we collected a comprehensive set of relevant information from our patient cohort. This information encompassed various factors such as age, sex, date of accident, concomitant injuries, pre-existing comorbidities, and treatment details. Specifically, we recorded the type of treatment administered, distinguishing between conservative management and surgical interventions involving plate osteosynthesis or intramedullary nailing.

To assess the presence and impact of comorbidities, we utilised the Charlson Comorbidity Index (CCI) as a standardised measure. This allowed us to evaluate the overall burden of chronic conditions in our patient population. In terms of fracture-related data, we employed X-rays of the affected shoulder taken in true anteroposterior and Y-view orientations. These radiographs were subjected to a thorough radiological evaluation using the widely accepted Neer classification system [11]. This classification system enabled us to categorise the fractures based on specific characteristics, facilitating a standardised approach to analysing and interpreting the data.

Following a minimum follow-up period of 5 years, we proactively contacted the members of our patient cohort through a combination of telephone and written correspondence. Through these communication channels, we gathered additional information pertaining to their outcomes, allowing us to evaluate the long-term effects of the chosen treatments. Furthermore, we diligently documented the aftercare scheme provided to each patient, ensuring a comprehensive overview of the care they received throughout the follow-up period.

### 2.2. Statistical Analysis

Statistical analysis was carried out using SPSS software package version 25 (SPSS Inc., Chicago, IL, USA). The chi-square independence test was performed to compare categorical variables. The independent *t*-test was used to compare continuous variables after determining that all variables are normally distributed (Kolmogorov–Smirnov normality test). *p*-values < 0.05 were considered significant. All graphs are displayed with mean values and 95% confidence intervals. To evaluate the independent mortality risk factors in our patient collective, we performed a multivariate binary logistic regression model. The influence of relevant known confounders (age, sex, treatment, comorbidities, and concomitant injuries) on mortality was analysed. All factors were included in logistic regression analysis, and *p*-values and odds ratios (ORs) for each factor were calculated, as well as the corresponding 95% confidence intervals (CIs).

## 3. Results

Our study included a total of 383 patients, 240 of whom were female, and 143 were male. The average age of the patients in our cohort was 72 ± 16 years. These patients met the specified inclusion and exclusion criteria, as illustrated in Figure 1. It is important to note that there were differences in the age distribution between the respondent and non-respondent groups. The respondent group had a higher mean age of 74 ± 16 years, while the non-respondent group had a lower mean age of 67 ± 16 years. The mean duration of follow-up for our patient collective was 10.5 ± 3.2 years, with a minimum follow-up period of 5 years. Among the total of 240 female patients, 188 responded to our survey, while 52 did not provide a response. In the case of male patients, 100 were part of the respondent group, while 43 did not respond. When analysing fracture classifications, we found that Neer type 4 fractures were the most frequently observed in the respondent group, with a total of 132 cases. In contrast, the non-respondent group had a higher prevalence of Neer type 5 fractures, with a total of 42 cases. Detailed information regarding the fracture classification can be found in Table 1.

Table 1 shows that the patient cohort is equally distributed, with no significant differences in responders and non-responders in regard to age, sex, and fracture type according to Neer’s classification and treatment option. Notably, 107 patients were treated conservatively, and 276 of the operatively treated patients either underwent plate osteosynthesis (194) or intramedullary nailing (82). Additionally, 77 of the non-surgically treated patients and 211 of the surgically treated patients responded in comparison to 30 non-surgically and 65 surgically treated patients who were non-responders.

**Figure 2:**
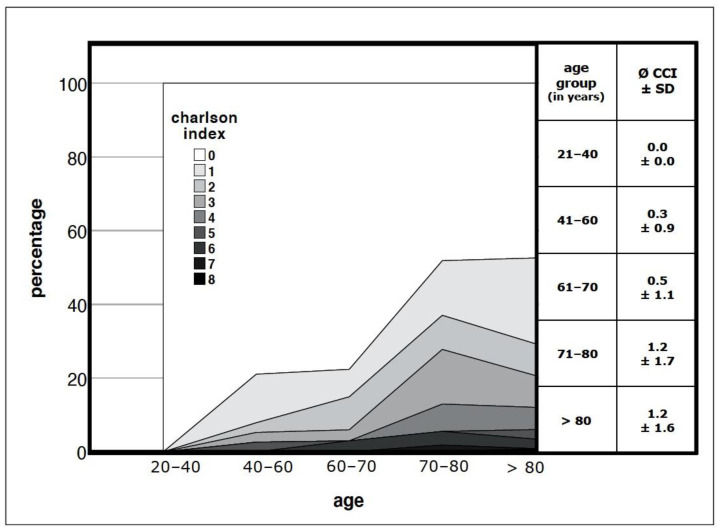
Analysis of the comorbidities in the presented patient collective: Correlation of a higher Charlson Comorbidity Index (CCI) with a higher age; *p* = 0.000; SD = standard deviation.

To analyse our patient collective further, we assessed pre-existing comorbidities according to the Charlson Comorbidity Index (CCI). For each patient, the CCI was analysed (Ø = 0.83 ± 1.45) and age-adjusted (Ø = 1.89 ± 1.46). To differentiate between patients with severe pre-existing conditions and those with moderate or no comorbidities, we subdivided our collective into groups of CCI of 0–2 (87.5%) and a CCI higher than 2 (12.5%). Our results clearly indicated that older patients are associated with a higher CCI (as shown in Figure 2) and thus have more comorbidities.

**Figure 3:**
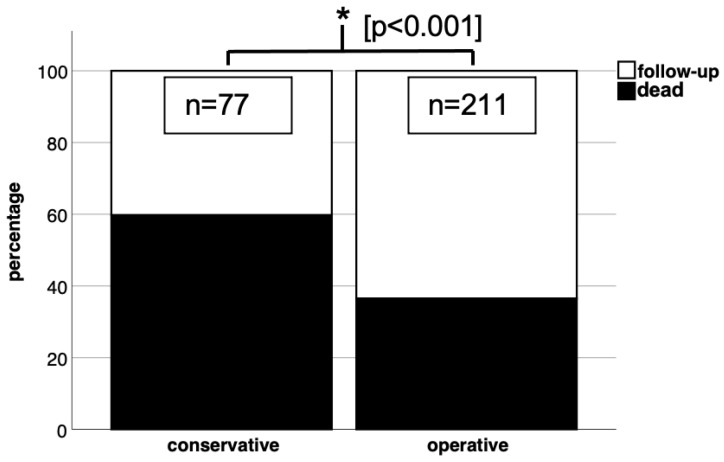
Conservative treatment in our patient collective showed significant results (*p* < 0.05 in chi-square test) with a higher mortality rate than surgical treatment.

**Figure 4:**
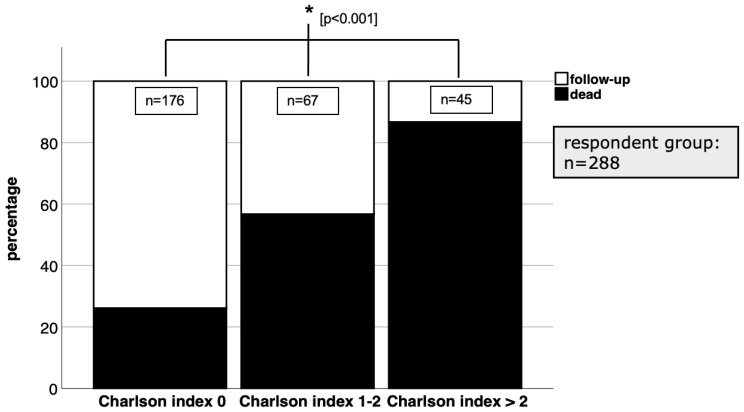
With a CCI of more than 2, mortality was significantly (*p* < 0.05 in chi-square test) higher than with lower CCI values.

**Figure 5:**
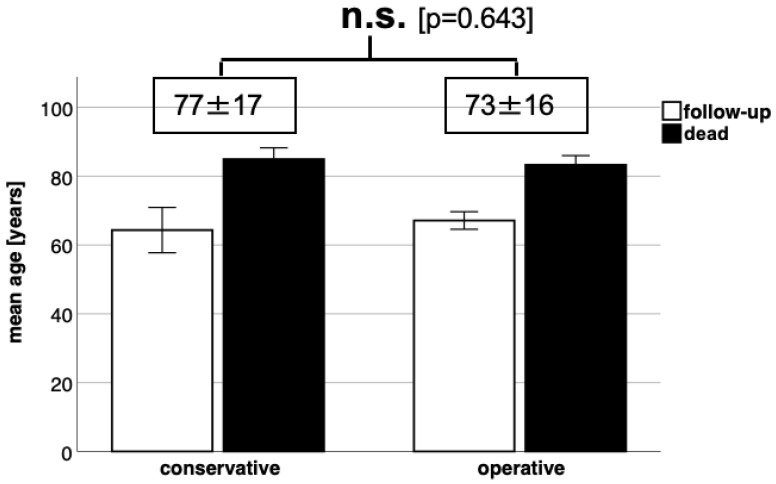
Comparison of the conservatively and surgically treated patients analysed according to mean age for death or follow-up group with no significant differences.

In our respondent patient group, the overall mortality rate was found to be 43.8% (126 out of 288 patients). The presence of concomitant injuries, totalling 83 cases, did not have a significant impact on the mortality rate (*p* > 0.05). However, when we categorised the patients into two treatment groups, namely conservative and surgical, and compared their respective death rates, we observed a significantly higher mortality rate in the conservatively treated group (Figure 3). Furthermore, our analysis revealed significant results that indicate a higher mortality rate among patients with comorbidities than among those without a history of chronic diseases (Figure 4). In the subgroup analysis, we specifically examined the mean age of patients in relation to the follow-up and death groups within the conservative and surgical treatment categories. However, no statistically relevant results were found in this analysis (Figure 5).

### Predictors of Death after Proximal Humerus Fractures

We evaluated independent mortality risk factors in proximal humerus fractures for our patient collective and found that mortality was increased by 10% per life year and was 3.9 times higher for male patients and 3.4 times higher for those who received conservative treatment (Table 2). The strongest predictor of death was for patients with severe comorbidities, which we defined as a CCI of more than 2—here, we found a 20 times higher risk for mortality.

Mortality in our patient collective was associated with the following risk factors:

A 1.1 times increased risk per life year;
A 3.9 times increased risk for men;
A 3.5 times increased risk with few comorbidities (CCI 1–2);
A 20 times increased risk with serious comorbidities (CCI >2);
A 3.4 times increased risk for conservative treatment.

## 4. Discussion

The key findings of the present study are as follows:

- Mortality in our patient collective was significantly higher for patients with a Charlson Comorbidity Index of higher than 2;
- Conservative treatment for PHFs correlated with a higher mortality rate.

The primary focus of this study was to examine the mortality rate following proximal humerus fractures (PHFs) and identify the patient-related factors that could serve as potential predictors of death. Notably, this study stands out due to its large study population, encompassing a total of 383 patients, and its extensive long-term follow-up period, with an average follow-up duration of approximately 10.5 years. These notable characteristics contribute to the robustness and reliability of our findings.

One key finding of our investigation was the identification of a heightened mortality risk among patients with multiple comorbidities, those who received conservative treatment, and male patients. These factors emerged as significant predictors of mortality in the context of PHFs. The association between comorbidities and mortality underscores the importance of considering the overall health status of patients when assessing their prognosis and potential outcomes. Similarly, the impact of treatment choice on mortality rates highlights the importance of selecting the most appropriate treatment approach based on individual patient factors and characteristics. The observed gender disparity in mortality rates highlights the need for gender-specific considerations in managing and predicting outcomes for patients with PHFs.

Despite the strengths of this study, certain limitations should be acknowledged. One limitation is the relatively low response rate among patients, which could potentially introduce a bias in the data. The patients’ age could be a relevant factor contributing to the lower response rate. It is important to consider the potential impact of non-response bias when interpreting the results, as it may influence the generalizability of our findings to the broader patient population.

Additionally, another limitation is the lack of detailed information regarding the circumstances of death for patients who did not survive. The absence of such information hinders a comprehensive understanding of the factors leading to mortality in PHF cases. Further research and data collection should aim to address this limitation and provide a more comprehensive picture of the contributing factors and mechanisms leading to mortality.

The substantial study population and long-term follow-up duration enhance the reliability and significance of our findings. However, the low response rate among patients and the limited information on the circumstances of death should be acknowledged as limitations. Future studies should address these limitations to further advance our understanding of mortality risks and outcomes associated with PHFs.

An additional limitation that warrants discussion is the exclusion of patients who underwent arthroplasty as their primary fracture treatment. This exclusion was implemented to mitigate potential confounding factors, as arthroplasty often involves progressive aftercare schemes [12]. However, it is important to note that the focus of this study was primarily on comparing different reconstructive treatment options for the humeral head. To facilitate this comparison, we utilised Neer’s classification system, a widely accepted framework for categorising proximal humerus fractures. Nevertheless, it is important to mention that there is ongoing controversy surrounding the optimal treatment approaches for humerus head fractures, which is evidenced by the wide range of therapeutic strategies employed [13,14].

It is relevant to state that Neer’s classification system, although widely used, does not provide information regarding the risk of humeral head necrosis, nor is it based on indications for further therapy [15]. Thus, the current classification system does not fully encompass fracture- and patient-related factors that could inform treatment decisions. Developing a classification system that considers these factors would provide a more solid foundation for future discussions and decision making regarding treatment options for proximal humerus fractures.

Considering these limitations, future research should aim to address these gaps and challenges. Refining the classification system by incorporating additional factors such as the risk of humeral head necrosis and indications for further therapy would contribute to a more comprehensive understanding of these types of fractures and inform tailored treatment approaches. Moreover, conducting studies that encompass a broader range of treatment options and consider the long-term outcomes and functional recovery of patients would provide valuable insights into optimising treatment strategies for proximal humerus fractures.

To advance the field, further research should address these limitations and focus on developing a comprehensive classification system that incorporates fracture- and patient-related factors to guide treatment decisions for proximal humerus fractures.

A notable study worth mentioning is a large-scale multicentre randomised controlled trial conducted in the United Kingdom [8,16]. This study involved 250 patients and included follow-up assessments at 2 and 5 years, among other time points. The findings from this trial revealed no significant differences in functional outcomes and quality of life between patients treated conservatively and those who underwent surgical interventions for proximal humerus fractures [8]. These results challenge the widespread assumption that surgical treatment offers superior benefits over conservative management.

When considering osteosynthetic procedures, it is crucial to weigh the potential need for material removal, especially in older patients. The possibility of subsequent material removal necessitates a careful evaluation of the treatment indication, particularly in the context of advanced age. Complications following osteosynthetic procedures for proximal humerus fractures have been frequently reported [17,18]. Additionally, long-term follow-up studies have failed to demonstrate the superiority of surgical interventions in the treatment of proximal humerus fractures [8,16]. These findings raise the fundamental question of when surgical therapy for proximal humerus fractures should be considered, given the absence of clear evidence supporting its overall superiority.

These findings challenge the prevailing treatment paradigm and suggest that a more selective approach to surgical interventions may be warranted. Factors such as patient characteristics, fracture complexity, functional demands, and surgeon experience should be carefully evaluated when determining the most appropriate treatment strategy for proximal humerus fractures. Further research is needed to elucidate the specific circumstances in which surgical intervention provides significant advantages over conservative management, as well as to identify the potential predictors of favourable outcomes following surgical treatment.

The high rate of complications associated with osteosynthetic procedures and the lack of clear superiority of surgical treatment in long-term follow-up studies raise questions about the indications for surgical therapy in proximal humerus fractures. A more selective and individualised approach to treatment decisions, considering various patient and fracture-related factors, is warranted. Further investigations are necessary to identify specific criteria for surgical intervention and to optimise treatment strategies for proximal humerus fractures. Regarding mortality, the British ProFHER study showed that mortality increased in the group of surgically treated patients [8].

A recent study conducted in Spain, which focused on mortality risk factors in proximal humerus fractures (PHFs), exhibited findings that aligned with the results from the ProFHER trial in the UK [8,19]. Specifically, they discovered a significantly higher mortality rate among patients who underwent surgical treatment and presented with severe comorbidities than those individuals who received conservative treatment and had severe comorbidities [19]. Interestingly, these results appear contradictory to our own findings derived from the binary logistic regression model utilised in our study. Our analysis indicated a notably elevated risk of mortality, approximately 3.4 times higher, among patients treated conservatively for PHFs. In the Spanish study as well as in our study, it is not possible to clearly differentiate whether the indication was achieved based on a patient’s possible desolate situation or whether the performed therapy was the determining factor for the mortality risk, because of the nature of a retrospective patient collective. 

Nonetheless, it is reasonable to hypothesise that the limited independence resulting from prolonged immobilisation in conservative treatment [20,21] may be associated with increased mortality in elderly patients [22]. Therefore, it is crucial to carefully evaluate the specific challenges faced by the elderly population and individuals with comorbidities, as these factors should significantly influence our treatment strategies. Looking ahead, the projected demographic shift toward an older population [23] suggests that proximal humerus fractures (PHFs) will become even more prevalent in the near future. Consequently, inactivity and the loss of independence associated with PHFs will become increasingly pertinent issues for our healthcare system. This realisation underscores the urgent need for a more comprehensive research approach to address the current and forthcoming questions surrounding PHF therapy.

To effectively manage PHFs and mitigate the potential adverse outcomes associated with conservative treatment, further studies are imperative. These studies should focus on elucidating optimal rehabilitation strategies that promote functional recovery and minimise the negative impacts of immobilisation. Moreover, investigations into innovative treatment modalities and interventions tailored to the unique needs of elderly patients and those with comorbidities are warranted. By dedicating more research efforts to PHFs, we can develop evidence-based guidelines and treatment algorithms that account for the specific challenges faced by different patient populations. This will enable us to provide more targeted, personalised care and mitigate the long-term consequences of PHFs.

Recognising the potential association between limited independence and increased mortality in conservative treatment, particularly in elderly patients, underscores the importance of considering the challenges faced by these individuals in our treatment strategies. With the anticipated rise in the elderly population, coupled with the escalating burden of PHF-related inactivity and loss of independence, it is imperative to undertake additional studies to address the current and emerging questions surrounding PHF therapy. The findings of the current study provide compelling evidence regarding the most significant independent predictors of mortality in patients with proximal humerus fractures within our patient cohort. Specifically, male sex, conservative treatment, and the presence of severe comorbidities, as indicated by a Charlson Comorbidity Index exceeding 2, emerged as the most relevant factors associated with an increased risk of death. These findings are particularly noteworthy considering the demographic shift toward an aging population. As the population ages, both patient-related and fracture-related factors should be given heightened consideration when making treatment decisions for proximal humerus fractures. The influence of these factors on treatment choice is expected to become increasingly important in the coming years.

By identifying the male sex, conservative treatment, and severe comorbidities as the prominent predictors of mortality in PHFs, this study underscores the importance of comprehensive patient assessment and individualised treatment approaches. Understanding the impact of these factors on patient outcomes is essential for optimising treatment strategies and improving patient care.

As the field progresses, it is imperative to continue exploring the intricate relationship between patient- and fracture-associated factors in the context of PHFs. Ongoing research should delve into the mechanisms underlying these associations and further refine our understanding of their implications for treatment decisions.

All in all, this study highlights the key predictors of mortality in patients with PHFs, emphasising the male sex, conservative treatment, and the presence of severe comorbidities. In an aging population, it is essential to consider the increasing influence of patient- and fracture-associated factors when determining the most appropriate treatment strategy. By integrating these factors into our decision-making process, we can optimise patient outcomes and enhance the overall quality of care for individuals with proximal humerus fractures.

## 5. Conclusions

In conclusion, our study findings highlight the significant independent predictors of mortality in patients with proximal humerus fractures (PHFs) within our patient collective. Male sex, conservative treatment, and severe comorbidities (Charlson Comorbidity Index > 2) emerged as the most relevant factors associated with increased risk of death. As the population continues to age, it becomes imperative to consider both patient- and fracture-related factors when making treatment decisions for PHF patients. This study underscores the need for a comprehensive approach that integrates these factors to optimise treatment outcomes in this population.

## Figures and Tables

**Table 1:** Patient cohort subdivided into respondent and non-respondent groups.

	Respondent Group		Non-Respondent Group	Total	*p*-Value
*n*	288		95	383	
age [*n*]	74 ± 16		67 ± 16	72 ± 16	0.536
sex [*n*]	♂ = 100 ♀ = 188		♂ = 43 ♀ = 52	♂ = 143 ♀ = 240	0.065
fracture classification [n]	Neer type 2	4	1	5	0.098
	Neer type 3	31	15	46	
	Neer type 4	132	33	165	
	Neer type 5	96	42	138	
	Neer type 6	25	4	29	
therapy [*n*] conservative	77		30	107	0.362
operative	211		65	276	
	plate	151	43	194	0.404
	intramedullary nail	60	22	82	

**Table 2:** Odds ratios (ORs) of relevant mortality risk factors in PHFs; SD = standard deviation; 95% CI = 95% confidence interval; * = significant.

Risk Factors	SD	*p*-Value	OR	95% CI	
				Lower Limit	Upper Limit
age per year	0.017	0.000	1.132 *	1.094	1.171
male sex	0.43	0.002	3.928 *	1.680	9.186
conservative treatment	0.394	0.002	3.431 *	1.584	7.433
concomitant injuries	0.376	0.805	1.097	0.525	2.291
few comorbidities (CCI 1-2)	0.376	0.001	3.540 *	1.695	7.393
severe comorbidities (CCI > 2)	0.581	0.000	20.383 *	6.530	63.627

## Data Availability

If required, our data can be submitted.

## References

[1] Rupp M., Walter N., Pfeifer C., Lang S., Kerschbaum M., Krutsch W., Baumann F., Alt V. (2021). The Incidence of Fractures Among the Adult Population of Germany—An Analysis From 2009 through 2019. Dtsch. Arztebl. Int..

[2] Du S., Ye J., Chen H., Li X., Lin Q. (2017). Interventions for Treating 3- or 4-part proximal humeral fractures in elderly patient: A network meta-analysis of randomized controlled trials. Int. J. Surg..

[3] McLean A.S., Price N., Graves S., Hatton A., Taylor F.J. (2019). Nationwide trends in management of proximal humeral fractures: An analysis of 77,966 cases from 2008 to 2017. J. Shoulder Elb. Surg..

[4] Klug A., Gramlich Y., Wincheringer D., Schmidt-Horlohé K., Hoffmann R. (2019). Trends in surgical management of proximal humeral fractures in adults: A nationwide study of records in Germany from 2007 to 2016. Trauma Surg..

[5] Hao K.A., Patch D.A., Reed L.A., Spitler C.A., Horneff J.G., Ahn J., Strelzow J.A., Hebert-Davies J., Little M.T., Krause P.C. (2021). Factors influencing surgical management of proximal humerus fractures: Do shoulder and trauma surgeons differ?. J. Shoulder Elb. Surg..

[6] Wu L., Jiang Y., Cao X., Meng X. (2021). Efficacies and complications of internal fixations with PHILOS plate and intramedullary Multiloc^®^ nails in the surgical treatment of proximal humerus fractures. Am. J. Transl. Res..

[7] Fraser A.N., Bjørdal J., Wagle T.M., Karlberg A.C., Lien O.A., Eilertsen L., Mader K., Apold H., Larsen L.B., Madsen J.E. (2020). Reverse Shoulder Arthroplasty Is Superior to Plate Fixation at 2 Years for Displaced Proximal Humeral Fractures in the Elderly: A Multicenter Randomized Controlled Trial. J. Bone Jt. Surg..

[8] Rangan A., Handoll H., Brealey S., Jefferson L., Keding A., Martin B.C., Goodchild L., Chuang L.-H., Hewitt C., Torgerson D. (2015). Surgical vs nonsurgical treatment of adults with displaced fractures of the proximal humerus: The PROFHER randomized clinical trial. JAMA.

[9] Marks L., Pass B., Knobe M., Volland R., Eschbach D., Lendemans S., Aigner R., Schoeneberg C. (2021). Registry for Geriatric Trauma. Quality of life, walking ability and change of living situation after trochanteric femur fracture in geriatric patients-Comparison between sliding hip screw and cephalomedullary nails from the registry for geriatric trauma. Injury.

[10] Vazquez O., Gamulin A., Hannouche D., Belaieff W. (2021). Osteosynthesis of non-displaced femoral neck fractures in the elderly population using the femoral neck system (FNS): Short-term clinical and radiological outcomes. J. Orthop. Surg. Res..

[11] Neer C.S. (1970). Displaced proximal humeral fractures. I. Classification and evaluation. J. Bone Jt. Surg. Am..

[12] Boudreau S., Boudreau E., Higgins L.D., Wilcox R.B. (2007). Rehabilitation following reverse total shoulder arthroplasty. J. Orthop. Sports Phys. Ther..

[13] Mease S.J., Kraeutler M.J., Gonzales-Luna D.C., Gregory J.M., Gardner M.J., Choo A.M. (2021). Current Controversies in the Treatment of Geriatric Proximal Humeral Fractures. J. Bone Jt. Surg..

[14] Schumaier A., Grawe B. (2018). Proximal Humerus Fractures: Evaluation and Management in the Elderly Patient. Geriatr. Orthop. Surg. Rehabil..

[15] Neer C.S. (2002). Four-segment classification of proximal humeral fractures: Purpose and reliable use. J. Shoulder Elbow Surg..

[16] Handoll H., Brealey S., Rangan A., Keding A., Corbacho B., Jefferson L., Chuang L.-H., Goodchild L., Hewitt C., Torgerson D. (2015). The ProFHER (PROximal Fracture of the Humerus: Evaluation by Randomisation) trial—A pragmatic multicentre randomised controlled trial evaluating the clinical effectiveness and cost-effectiveness of surgical compared with non-surgical treatment for proximal fracture of the humerus in adults. Health Technol. Assess..

[17] Oldrini L.M., Feltri P., Albanese J., Marbach F., Filardo G., Candrian C. (2022). PHILOS Synthesis for Proximal Humerus Fractures Has High Complications and Reintervention Rates: A Systematic Review and Meta-Analysis. Life.

[18] Plath J.E., Kerschbaum C., Seebauer T., Holz R., Henderson D.J.H., Förch S., Mayr E. (2019). Locking nail versus locking plate for proximal humeral fracture fixation in an elderly population: A prospective randomised controlled trial. BMC Musculoskelet. Disord..

[19] Garcia-Reza A., Dominguez-Prado D.M., Iglesias-Nuñez C., Alvarez-Alvarez L., Hernandez-Gonzalez B., Balvis-Balvis P., Fernandez-Fernandez D., Castro-Menendez M. (2021). Analysis of predictors of mortality after surgical and non-surgical management in proximal humerus fractures. J. Orthop. Traumatol..

[20] Van den Broek C.M., van den Besselaar M., Coenen J.M., Vegt P.A. (2006). Displaced proximal humeral fractures: Intramedullary nailing versus conservative treatment. Arch. Orthop. Trauma Surg..

[21] Torrens C., Corrales M., Vilà G., Santana F., Cáceres E. (2011). Functional and quality-of-life results of displaced and nondisplaced proximal humeral fractures treated conservatively. J. Orthop. Trauma.

[22] Rotman D., Giladi O., Senderey A.B., Dallich A., Dolkart O., Kadar A., Maman E., Chechik O. (2018). Mortality After Complex Displaced Proximal Humerus Fractures in Elderly Patients: Conservative Versus Operative Treatment With Reverse Total Shoulder Arthroplasty. Geriatr. Orthop. Surg. Rehabil..

[23] Nowossadeck E. (2012). Population aging and hospitalization for chronic disease in Germany. Dtsch. Arztebl. Int..

